# Factors determining late antenatal care booking and the content of care among pregnant mother attending antenatal care services in East Wollega administrative zone, West Ethiopia

**DOI:** 10.11604/pamj.2017.27.184.10926

**Published:** 2017-07-07

**Authors:** Eyasu Ejeta, Regea Dabsu, Olifan Zewdie, Elias Merdassa

**Affiliations:** 1 Department of Medical Laboratory Sciences, College of Medical and Health Sciences, Wollega University, Nekemte, Ethiopia; 2 Department of Medical Laboratory Sciences and Pathology, College Health Sciences, Jimma Univerity, Jimma, Ethiopia; 3 Department of Public Health College of Medical and Health Sciences, Wollega University, Nekemte, Ethiopia

**Keywords:** Pregnant women, ANC initiation, factors, Ethiopia

## Abstract

**Introduction:**

Antenatal care (ANC) is important for both maternal and fetal health. However, the existing evidence from developing countries indicates that most pregnant women attending ANC in their late pregnancy. Little is known about the factors determining ANC booking and the content of care among pregnant women in West part of Ethiopia. Therefore, the present study was conducted to identify factors determining late ANC booking and the content of care among pregnant mother attending antenatal care services in East Wollega administrative zone, West Ethiopia.

**Methods:**

Institutional based cross-sectional study was conducted from July to September, 2014 among 421 pregnant women's attending ANC services in purposively selected health facilities, East Wollega zone, Ethiopia. The pretested-structured questionnaires were used to collect socio-demographic data and predictor factors of late initiation of ANC services. Five trained nurse working at ANC clinic at each health institution administered the questionnaire. The collected data was analysed using SPSS version 20.

**Results:**

The prevalence of late ANC booking was 81.5% (343/421) in the study area. Being from Oromo ethnic group (AOR 4.27, (95% CI, 1.48-12.33)), maternal age equal or more than 25 year old (AOR 3.09 (95% CI, 1.53-6.27)), second trimester (AOR 6.05(95% CI, 3.08-11.88)) and third trimester (AOR 7.97 (95% CI, 3.92-16.23)) were main factors identified as contributing (favoring factors) for the likely occurrence of late booking for ANC whereas; monthly income more than and/or equal to 15000 Ethiopian birrs (AOR 0.38 (95% CI, 0.18-084)) were factors compromising (decreasing) the chances for late attendance for the services among the pregnant women.

**Conclusion:**

Late ANC initiation is high in the study area despite the services is provided free of charge. Hence, it is important to provide health education on the timing of ANC among women with reproductive age. Community's awareness on importance of receiving early ANC also needs to be promoted.

## Introduction

Worldwide, pregnancy and childbirth related complications are contributing to significant public health problems. The burden is high among developing countries which carries 99% of maternal deaths. Of these, more than half of these deaths occurred in sub-Saharan Africa including Ethiopia [1–3]. Antenatal care (ANC) is one of the four pillars of the initiative for safe motherhood [4]. The main objectives of ANC are: prevention and treatment of obstetric complications, preparation for emergencies, family planning, meeting nutritional, social, emotional needs for pregnant woman, including care and nutrition of the newborn [5]. Women attending ANC visits receive sufficient evidence-based clinical interventions, such as tetanus toxoid immunization, deworming, iron and folic acid supplements, counseling on maternal health, emergency preparedness, management of sexually transmitted infections, administration of antiretroviral therapy in HIV-positive women, supply of essential information about improved hygienic practices and the risks associated with pregnancy and childbirth [6]. The World Health Organization (WHO) recommends that pregnant women in developing countries initiate early prenatal care before the end of the fourth month of pregnancy [7]. ANC for the first trimester is fundamental and decisive to identify and evaluate the risk factors usually present before pregnancy [7]. According to different studies done previously, there are factors associated with late entry to ANC, these include place of residence, ethnicity, age, education, employment status, parity, intention to get pregnant, use of contraceptive methods, economic status, health insurance and travel time [8–13]. However, despite concerted efforts to scale-up ANC services in Ethiopia, the coverage and uptake of the service by the pregnant women remains low and unevenly distributed as the recent demographic and health survey report [3]. There is surprisingly little information on the challenges and obstacles to ANC initiation in the present selected studies area. Therefore, the aim of this study was to assess the factors associated with ANC booking and the content of care among pregnant mother attending antenatal care services in East Wollega administrative zone, West Ethiopia. Such information provides evidence for the identification of those factors contributing to the poor implementation of ANC services and fills the policy gaps towards improving ANC services.

## Methods

**Study setting and design**: The study was conducted in East Wollega administrative zone, West Ethiopia which located at 328km distance from capital city of Addis Ababa. Total population of the study area was 1,230,402, with 1:1 ratio male to female. The zone has seventeen administrative districts including Nekemte town which is the capital city of the zone. In the zone there are 2 hospitals, 59 health centers and 297 health posts with the zonal health service coverage of 96% [14]. These all health facilities provide ANC service for all pregnant women visiting these institutions free of charge. Cross sectional descriptive study design was conducted among purposively selected five health facilities (Nekemte referral hospital, Nekemte health center, Getema health center, Arjo Gudetu and Sire health center) in four districts of East Wollega administrative zone, Ethiopia between July to September, 2014.

**Study population and participant sampling**: The study population was all pregnant women attending antenatal care clinic (ANC) at the selected five health facilities during the study period for ANC services. Pregnant women who came for ANC service at these health institutions for the current pregnancy and residents of the zone were included in this study. Pregnant women aged 16-36 years were eligible for participation in the study. A total of 421 pregnant women attending antenatal acre clinic (ANC) at the study institutions, were recruited and interviewed using structured questionnaire at the time of exit.

**Data collection procedures**: The questionnaire used in the data collection was derived from related questions used in similar studies. The questionnaire was also pretested on 5% of the total sample size in the ANC clinic at Gutin health center, Ethiopia. The questionnaire was then assessed for its clarity and completeness. Some skip patterns were corrected, questions difficult to ask were rephrased and the consent form was modified. Five trained nurses working at ANC clinic at each health institution administered the questionnaire.

**Data processing and statistical analysis**: Data were analyzed by SPSS version 20 for windows. Backward logistic regression model was used to control for the possible cofounders. Finally, multivariate logistic regression analysis was undertaken by including factors found to be significant or marginally at P-value < 0.25 in binary logistic analysis. Statistical significance was evaluated at 95% levels of significance. Those variables with p-value < 0.05 on the final model were identified as the associated factors for ANC late initiation time. If a mother came for ANC before or at 16 weeks of gestation for the first time during the pregnancy; she was considered as having early booking visit (within the recommended time) unless considered as late attendance.

**Ethical clearance**: Letter of ethical clearance was obtained from the ethical review board of Wollega University. The health centers included in this study were asked permission using formal letters from the university. Informed verbal consent was obtained from the study participants. Privacy of clients and all information related to study participants was maintained confidential.

## Results

**Socio-demographic of characteristics**: A total of 421 pregnant women were participated in this study. More than half 254(60.3%) the respondents were urban dwellers; majority of them were from oromo 380 (90.3%) ethnic group whereas 250(59.4%) were protestant followers. Majority 285(67.7%) of the respondents were in the age group of 18-24 years, with the mean age of 22 years. Great majority 407(96.7%) were married. Around 352(83.6%) of the mothers were housewives and 107(25.4%) illiterate (Table 1). Few mothers 66(15.7%) were booked for ANC in first trimester, about half 201(47.7%) of them were Multiparous and 82(19.5) had large family size (Table 1).

**Table 1:** Prevalence of late initiation ANC and content care among pregnant women attending antenatal care at East Wollega administrative zones, Ethiopian, 2014 (n=421)

Socio-demographic variables	ANC imitation		Total n (%)
	Late n (%)	Early n (%)	
**Residence**			
Urban	204(80.3)	50(19.7)	254(60.3)
Rural	139(83.2)	28(16.8)	167(39.7)
**Ethnicity**			
Amhara	15(68.2)	7(31.8)	22(5.2)
Other*	16(84.2)	3(15.8)	19(4.5)
Oromo	312(82.1)	68(17.9)	380(90.3)
**Religion**			
Orthodox	95(79.2)	25(20.8)	120(28.5)
Muslim	38(82.6)	8(17.4)	46(10.9)
Protestant	206(82.4)	44(17.6)	250(59.4)
Other	4(80)	1(20)	5(1.2)
**Occupation**			
Employed	50(72.5)	19(27.5)	69(16.4)
House wife	293(83.2)	59(16.8)	352(83.6)
**Monthly income**			
<500	177(85.5)	30(14.5)	207(49.2)
501-1499	119(79.3)	31(20.7)	150(35.6)
>1500	47(73.4)	17(26.6)	64(15.2)
**Age( Mean =22.72)**			
18-24	220(77.2)	65(22.8)	285(67.7)
25-31	110(90.9)	11(9.1)	121(28.7)
>31	13(86.7)	2(13.3)	15(3.6)
**Education level**			
Illiterate	95(88.8)	12(11.2)	107(25.4)
Primary	122(80.8)	29(19.2)	151(35.9)
Secondary and above	126(77.3)	37(22.7)	163(38.7)
**Marital status**			
Married	332(81.6)	75(18.4)	407(96.7)
Other**	11(78.6)	3(21.4)	14(3.3)
**Gestational stage**			
1^st^ trimester	33(50.0)	33(50.0)	66(15.7)
2^nd^ trimester	160(86.5)	25(13.5)	185(43.9)
3^rd^ trimester	150(88.2)	20(11.8)	170(40.4)
**Parity**			
Uniparous	172(78.2)	48(21.8)	220(52.3)
Multiparous	171(85.1)	30(14.9)	201(47.7)
**Family size**			
<2	161(78.9)	43(21.1)	204(48.5)
3-4	107(79.3)	28(20.7)	135(32.1)
>5	75(91.5)	7(8.5)	82(19.5)
**Number of ANC follow up**			
One	170(81.0)	40(19.0)	210(49.9)
Two	113(85.0)	20(15.0)	133(31.6)
Three	43(81.1)	10(18.9)	53(12.6)
More than three	17(68.0)	8(32.0)	25(5.9)
**Study institutions**			
Nekemte Health Center	123(82.0)	27(18.0)	150(35.6)
Nekemte Referral Hospital	59(68.6)	27(31.4)	86(20.4)
Getema Health Center	23(79.3)	6(20.7)	29(6.9)
Sire Health Center	75(97.4)	2(2.6)	77(18.3)
ArjioGudatu Health Center	63(79.7)	16(20.3)	79(18.8)

* = Tigray, Gurage

** = divorced, widowed, 1$USD=21 Ethiopian birrs

**Figure 1:**
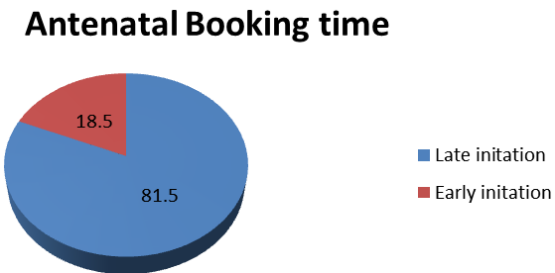
Antenatal care initiation time among pregnant women attending antenatal care at East Wollega administrative zones, Ethiopian, 2014 (n = 421)

**Figure 2:**
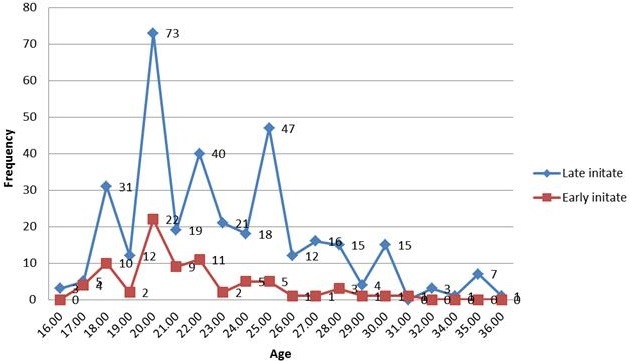
Age based distribution of ANC and content care among pregnant women attending antenatal care at East Wollega administrative zones, Ethiopian, 2014 (n = 421)

**Antenatal care initiation time**: Majority of the clients booked for antenatal care service lately 343 (81.5%) where as some 78(18.5%) booked earlier (Figure 1). There was an overall of higher prevalence of late ANC booking among rural residents (83.2%), Tigray and Gurage ethnic group (84.2%) and participants having lower average monthly income (85.5%). There was also high rate of late ANC booking among illiterate respondents (88.8%), multiparous women (85.1%), more than 25 years old women (90.4) and family having higher family size (91.5%) (Table 1). The prevalence of late ANC booking was increase with material age until 25 years old but decrease then after (Figure 2).

**Determining factor for late initiation of ANC and content of care among pregnant women**: After adjusting for socio-demographic and contextual factors; being from Oromo ethnic group [AOR 4.27, (95% CI, 1.48-12.33)], maternal age equal or more than 25 year old [AOR 3.09 (95% CI, 1.53-6.27)], second trimester [AOR 6.05(95% CI, 3.08-11.88)] and third trimester [AOR 7.97 (95% CI, 3.92-16.23)] were main factors identified as contributing (favoring factors) for the likely occurrence of late booking for ANC whereas; monthly income more than and/or equal to 15000 Ethiopian birrs [AOR 0.38 (95% CI, 0.18-084)] were factors compromising (decreasing) the chances for late attendance for the services among the pregnant women (Table 2).

**Table 2:** Factor associated with late initiation of ANC and content of care among pregnant women attending antenatal care at East Wollega administrative zones, Ethiopian, 2014 (n=421)

Variable	Total n (%)	Late ANC Initiation (%)	COR (CI)	P- value	AOR(CI)	P- Value
**Residence**						
Urban	254(60.3)	204(80.3)	1	-	-	-
Rural	167(39.7)	139(83.2)	1.22(0.73-2.03)	0.451	-	-
**Ethnicity**						
Amhara	22(5.2)	15(68.2)	1	-	-	-
Other*	19(4.5)	16(84.2)	2.48(0.54-1.43)	0.241	3.51(0.66-18.58)	0.139
Oromo	380(90.3)	312(82.1)	2.14(0.84-5.45)	0.110	4.27(1.48-12.33)	0.007*
**Religion**						
Orthodox	120(28.5)	95(79.2)	1	-	-	-
Muslim	46(10.9)	38(82.6)	1.25(0.52-3.01)	0.619	-	-
Protestant	250(59.4)	206(82.4)	1.23(0.71-2.13)	0.455	-	-
Other	5(1.2)	4(80)	1.05(0.11-9.84)	0.964	-	-
**Occupation**						
Employed	69(16.4)	50(72.5)	1	-	1	-
House wife	352(83.6)	293(83.2)	1.88(1.04-3.43)	0.037	1.88(0.95-3.71)	0.069
**Monthly income**						
<500	207(49.2)	177(85.5)	1	-	1	-
501-1499	150(35.6)	119(79.3)	0.65(0.37-1.13)	0.128	0.55(0.29-1.03)	0.063
>1500	64(15.2)	47(73.4)	0.47(0.24-0.92)	0.028	0.38(0.18-084	0.016*
**Age (Mean=22.72)**						
<25	285 (67.7)	220(77.2)	1	-	1	-
>25	136 (32.3)	123(90.4)	2.79(1.48-5.27)	0.002	3.09(1.53-6.27)	0.002*
**Education level**						
Illiterate	107(25.4)	95(88.8)	1	-	1	-
Primary	151(35.9)	122(80.8)	0.53(0.26-1.09)	0.087	0.57(0.25-1.30)	0.182
Secondary and above	163(38.7)	126(77.3)	0.43(0.21-0.87)	0.019	0.52(0.22-1.23)	0.135
**Marital status**						
Married	407(96.7)	332(81.6)	1	-	-	-
Other**	14(3.3)	11(78.6)	0.83(0.23-3.04)	0.777	-	-
**Gestational stage**						
1^st^ trimester	66(15.7)	33(50.0)	1	-	1	-
2^nd^ trimester	185(43.9)	160(86.5)	6.40(3.37-2.14)	0.000	6.05(3.08-11.88)	0.000*
3^rd^ trimester	170(40.4)	150(88.2)	7.50(3.83-14.7)	0.000	7.97(3.92-16.23)	0.000*
**Parity**						
Uniparous	220(52.3)	172(78.2)	1	-	1	-
Multiparous	201(47.7)	171(85.1)	1.59(0.96-2.63)	0.070	1.52(0.65-3.55)	0.331
**Family size**						
<2	204(48.5)	161(78.9)	1	-	1	-
3-4	135(32.1)	107(79.3)	1.02(0.59-1.74)	0.940	0.65(0.35-1.22)	0.185
>5	82(19.5)	75(91.5)	2.86(1.23-6.66)	0.015	1.37(0.52-3.57)	0.523
**knowledge of ANC**						
Yes	359(85.3)	294(81.9)	1	-	-	-
No	62(14.7)	49(79.0)	0.83(0.43-1.62)	0.593	-	-

COR, odds ratio; AOR, adjusted odds ratio; CI, confidence interval; 1, reference

## Discussion

Although the national and regional data on ANC attendance illustrate varying trends across sub-saharan Africa [15], good care during pregnancy is important for the health of the mother and the development of the unborn baby. According to WHO and the National Institute for Health and Clinical Excellence recommendations; every pregnant mother should start ANC booking with in the first fourth month of pregnancy [7]. However, 81.5% of the pregnant women were initiated ANC attendance after recommend period in this study. This is consistence with study conducted in Gamo Gofa Zone (Ethiopia) [11] and Ambo Town (Ethiopia) [16], but the proportion of pregnant mothers who booked within the recommended time was low in this study compared with the finding from Addis Ababa [17] and Gondar [18]; this observed difference could be due to the fact that, these two studies were conducted in big city of the country which might have better health awareness comparing with communities served at rural health facilities as the present study involve both urban and rural health institution. In this study, mothers who are aged 25 years and above were less likely to start ANC within the recommended period than those whose age was less than 25 years. This finding is supported by studies done in Gondar and Addis Ababa [17, 18]. This is possible that elder mothers women feel more confident after previous experience and feel that starting ANC early is not necessary. The presence high prevalence of late initiation of ANC among elder women in present studies supports this condition. Alternatively, young pregnant mothers might more likely be literate than elder mothers.

In general, level of knowledge, experiences, power to make decisions is increasing with age. Household income was one of the factors significantly associated with late antenatal care entry in this study. This result support several other studies done in Gamo Gofa Zone (Ethiopia) [11], Metekel zone (Ethiopia) [19] and Uganda [20] which showed that low monthly income is associated with increased odds of underutilizing antennal care services among pregnant mother. This could be attributed to the fact of better income might increase the ability to pay for transportation, health care services and other indirect costs. Mothers who book ANC early will have high likely to come for more number of follow-up. This could be attributed to the impact of information obtained from the health care provider in their first visit to properly utilize the ANC services. The evidence for an association between ethnicity and late or poor attendance for antenatal care may be slightly stronger than for social class as studies shown [21]. This is supported by the present studies. Further studies on the barriers to equitable access to antenatal care for women from different ethnic group would be valuable. This study has some limitations. One of the limitations is the study was a facility based cross-sectional study whose findings are not generalized to a general population. Moreover, the pregnant women who attend antenatal care at private health facilities were not included in the study.

## Conclusion

Our study showed that the late book for ANC was high in study area. This study indicated that low monthly income, maternal age, gestational stage and ethnicity of the women were factors associated with late first antenatal care booking. Hence, it is important to provide health education on the timing of ANC among women with reproductive age. Community's awareness on importance of receiving early ANC also needs to be promoted.

### is known about this topic

- Antenatal care (ANC) is one of the four pillars of the initiative for safe motherhood;
- High prevalence of late ANC booking among most pregnant women in developing countries;
- The coverage and uptake of ANC service by the pregnant women remains low and unevenly distributed in Ethiopia.

### What this study adds

- The prevalence of late ANC booking was high (81.5%) in the study area;
- Family income, maternal age, gestational stage and ethnicity of the mother were found to be significantly factor determining the late ANC booking in the area;
- The study shows the need of providing health education on the timing of ANC among women with reproductive age.

## Competing interests

The authors declare no competing interest.
